# Diagnostic parameters in native joint septic arthritis and development of a new evaluation score

**DOI:** 10.5194/jbji-11-175-2026

**Published:** 2026-03-23

**Authors:** Lorenz Huber, Hasan S. Aguicenoglu, Susanne Baertl, Julia Elisabeth Lenz, Kristina Gerhardinger, Frank Hanses, Florian Zeman, Nike Walter, Volker Alt, Markus Rupp

**Affiliations:** 1 Department for Trauma Surgery, University Hospital Regensburg, Franz-Josef-Strauss-Allee 11, 93053 Regensburg, Germany; 2 Department of Infection Prevention and Infectious Diseases, University Hospital Regensburg, Regensburg, Germany; 3 Department for Infection Control and Infectious Diseases, University Hospital Regensburg, Regensburg, Germany; 4 Center for Clinical Studies, University Hospital Regensburg, Regensburg, Germany; 5 Department of Psychosomatic Medicine, University Hospital Regensburg, Regensburg, Germany; 6 Department of Trauma-, Hand- and Reconstructive Surgery, Justus Liebig University Giessen, Giessen, Germany

## Abstract

**Introduction**: Septic arthritis (SA) is an orthopaedic emergency; delayed treatment endangers joint function and survival. Unlike periprosthetic infections, diagnostic criteria for native joints are poorly standardized. This study aimed to (1) evaluate diagnostic parameters including synovial white blood cell (sWBC) count, neutrophil percentage, serum C-reactive protein (CRP), and leukocyte count and (2) assess a new evaluation score combining these parameters. **Methods**: In a retrospective cohort study, cases of knee and shoulder SA treated at a German university hospital (2013–2022) were analysed. Parameters included synovial fluid analysis (sWBC, neutrophils), blood samples (CRP, leukocytes), and intraoperative cultures. Cut-offs for sWBC and neutrophils were determined using receiver-operating characteristic (ROC) analysis and the Youden index, comparing SA patients with non-infected controls. A new evaluation score for SA (Septic Arthritis Evaluation Score, SAES) was created: 2 points each for sWBC and neutrophils and 1 point each for CRP and leukocytes. **Results**: Complete data were available for 45 patients (71.4 % male, mean age 64.3 years). Knees were affected in 73.7 %, and shoulders were affected in 26.3 %. Median values were as follows: leukocytes – 11/nl, CRP – 158 mg L^−1^, sWBC – 42 910/ µ L, and neutrophils – 93.6 %. ROC analysis identified an optimal sWBC cut-off of 35 650/ µ L (sensitivity of 64.4 %, specificity of 87.8 %). The SAES showed higher discriminatory performance; with a threshold ≥ 3 points, sensitivity was 95.6 %, and specificity was 70.7 %. **Conclusions**: In this retrospective cohort, commonly used laboratory parameters for native joint SA showed limited discriminatory ability when applied individually. A newly developed composite score combining synovial and serum markers demonstrated higher sensitivity within this dataset. Prospective validation in larger cohorts is required before clinical application.

## 1 Introduction

Septic arthritis (SA) is an orthopaedic emergency that, if treated too late or inadequately, endangers the joint or even the life of the patient (Mathews et al., 2010). The annual incidence rate of SA of the native joint is 2–10 per 100 000 inhabitants in Europe and North America (Goldenberg, 1998; Ross, 2017). The knee is the most affected joint in SA, accounting for over 50 % of all infections (Gunnlaugsdóttir et al., 2022). Most joint infections are of hematogenous origin, followed by post-interventional and post-traumatic causes (Kemmerer et al., 2017; Kaandorp et al., 1997). Risk factors favouring the development of SA include diabetes mellitus, immunosuppression, and active malignancies (Shirtliff and Mader, 2002).

Patients usually present with pain, redness, and warmth of the joint. Nonspecific signs of infection such as fever or chills may be present (Carpenter et al., 2011). Therefore, a prompt and accurate diagnosis of SA is often challenging. Furthermore, leading orthopaedic and trauma societies have not yet established clear guidelines or algorithms for the diagnosis and treatment of SA. Additionally, existing recommendations for periprosthetic joint infections cannot be extrapolated to infections of native joints (Gramlich et al., 2020).

Serum inflammatory markers such as C-reactive protein (CRP), leukocyte count, and erythrocyte sedimentation rate (ESR) can provide valuable indications for the diagnosis of SA; however, they are not definitive on their own (Carpenter et al., 2011; Kemmerer et al., 2017). Another mandatory diagnostic step is the aspiration of the affected joint. On the one hand, it can be used for the detection of the causative pathogen; on the other hand, it is also helpful as an immediate diagnostic tool by performing a synovial fluid analysis, determining the synovial white blood cell count and cell differentiation. Massey et al. (2021) proposed a recommended leukocyte count cut-off of 33 000 cells/ µ L for patients who have not previously received antibiotic therapy (Massey et al., 2021), while other studies have reported thresholds of up to 50 000 leucocytes/ µ L (Turner et al., 2021). However, septic arthritis may also be present at substantially lower cell counts, particularly in critically ill or immunosuppressed patients (Gramlich et al., 2020; Gerlach, 2018). This wide range of reported cut-off values reflects a fundamental diagnostic trade-off: higher thresholds improve specificity but increase the risk of false-negative results, whereas lower thresholds enhance sensitivity at the expense of specificity.

Crystal-induced arthritis represents a major differential diagnosis of septic arthritis as both conditions may present with similar clinical and laboratory findings. Although the detection of crystals in synovial fluid supports a non-infectious etiology, crystal arthritis does not exclude concomitant infection, and false-negative crystal analysis has been reported (Baillet et al., 2019; Papanicolas et al., 2012).

Therefore, the aim of this study was to (1) assess the diagnostic performance of commonly used laboratory parameters, including sWBC count cut-offs and neutrophil percentage cut-offs, serum CRP, and serum leukocyte count and (2) based on these results, construct a composite evaluation score for SA and assess its performance in comparison with individual laboratory parameters.

## 2 Methods

### 2.1 Study cohort

This retrospective cohort analysis comprised all cases of SA treated between 2013 and 2022 at the University Hospital Regensburg, a German level-1 trauma centre with more than 35 000 emergency admissions per year. Cases were identified by examining discharge documents and associated medical records, which were screened by the International Classification of Disease (ICD)-10 diagnosis codes “M00.-, septic arthritis”. Epidemiological data were collected, such as age, sex, and clinical information on the included patients. In addition, co-morbidities and pre-existing conditions were reviewed, and the Charlson Comorbidity Index (CCI) was calculated (Charlson et al., 1987). Patients under 18 years of age and patients with pre-existing osteomyelitis and periprosthetic joint infections were excluded. Patients who had pre-existing antibiotic therapy or evidence of gout crystals in the synovial fluid analysis were also excluded to avoid distorting the results (Luo et al., 2020; Massey et al., 2021).

**Figure 1:**
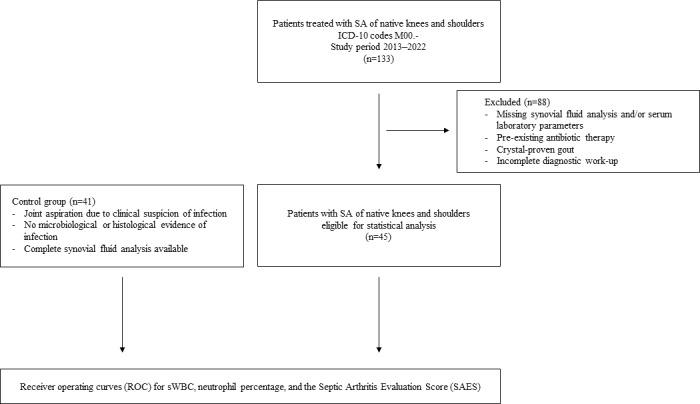
Flow diagram illustrating patient inclusion and exclusion. Of 133 patients with SA of native knees or shoulders, 45 patients with complete diagnostic data were included in the receiver-operating characteristic (ROC) analysis. A control group of 41 patients without evidence of infection was used for comparison. A total of 88 patients were excluded from ROC analysis due to incomplete diagnostic data.

For the calculation of the optimal cut-off value based on receiver-operating characteristic (ROC) analysis, a control group of patients without evidence of SA was defined. These patients underwent joint aspiration due to clinical suspicion of infection, which was subsequently excluded. The final diagnoses in this group were osteoarthritis ( n=17 , 41.5 %), acute traumatic joint effusion ( n=12 , 31.7 %), rheumatic inflammatory joint disease ( n=6 , 14.6 %), and other degenerative joint disorders ( n=5 , 12.2 %). Diagnostic aspirates of these patients yielded negative results. Only patients with SA of native knees and shoulders were included in the analysis, and the control group accordingly consisted exclusively of patients with synovial fluid samples from knee and shoulder joints. Patient flow is shown in Fig. 1.

The study was approved by the Ethics Committee of the University of Regensburg (approval no. 20-1681_1-104).

### 2.2 Definition of infection

A joint was considered to be infected if either (1) microbiological analysis of the joint aspirates, performed before surgery, or three to six tissue samples, which were collected intraoperatively, revealed a pathogen in at least one sample and/or (2) histological examination revealed features consistent with infection (e.g. acute neutrophilic infiltration, synovial hyperplasia, vascular congestion, tissue necrosis, or the presence of bacteria). Tissue samples were incubated for an extended period of 14 d to ensure the detection of low-virulence pathogens.

Clinical signs such as erythema, swelling, local warmth, and restricted joint function were not included in the diagnostic definition as these findings are non-specific and subject to considerable interobserver variability. This approach is consistent with established definitions for periprosthetic joint infection, such as those proposed by the European Bone and Joint Infection Society (McNally et al., 2021) and the Musculoskeletal Infection Society (Parvizi et al., 2018).

The joint aspirations were performed under sterile conditions in an outpatient operating room using an established test kit (Gramlich et al., 2022). The samples were subsequently subjected to synovial fluid analysis for synovial white blood cell count (sWBC), neutrophil percentage, gout crystals, and macroscopic properties such as colouration. The microbiological samples were tested in a paediatric blood culture system and incubated for an extended period of 14 d. Histopathological evaluation of the tissue samples was performed as part of routine clinical diagnostics. Histopathological samples were obtained exclusively during surgical procedures and were therefore only available in patients who underwent operative intervention. Patients in the control group were not routinely operated on; consequently, histological samples were only available in selected control cases when surgery was clinically indicated. Samples were assessed for features suggestive of infection, including acute neutrophilic infiltration, synovial hyperplasia, vascular congestion, tissue necrosis, and the presence of microorganisms. No quantitative or standardized scoring system was applied.

### 2.3 Septic Arthritis Evaluation Score

**Figure 2:**
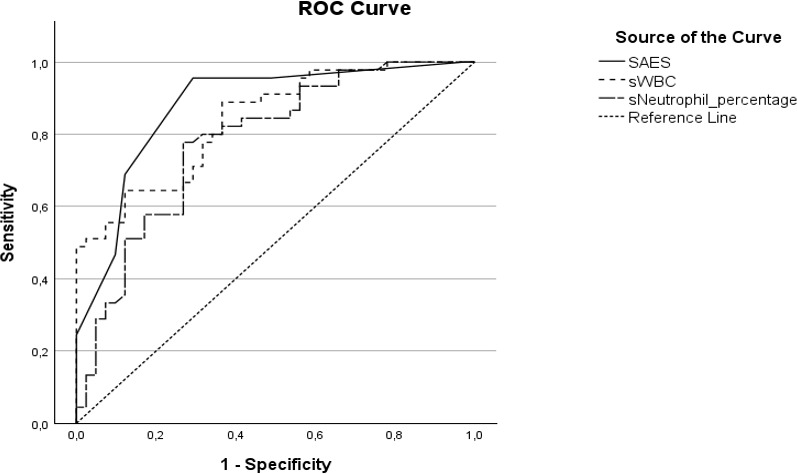
Receiver-operating characteristic (ROC) curves for Septic Arthritis Evaluation Score (SAES, solid line), synovial white blood cell count (sWBC, dotted line), and neutrophil percentage (dashed line). The area under the curve is 0.877 for SAES, 0.840 for sWBC, and 0.781 for synovial neutrophil percentage.

The Septic Arthritis Evaluation Score (SAES) was developed as an additional supportive tool to aid in the evaluation of whether septic arthritis may be present in clinically nonspecific cases. It was constructed post hoc based on the results of the parameter evaluation in this cohort. The score ranges from zero to six points, with points assigned for each of the following criteria: two points each for sWBC > 35 650 cells/ µ L and a neutrophil percentage > 90.6 %, identified as the best cut-off values in the ROC analysis of this cohort (see the Results section and Fig. 2). Elevated serum CRP > 5 mg L^−1^ and either leukocytosis > 12/nl or leukopenia < 4/nl, based on SIRS criteria, were also assigned one point (Baddam and Burns, 2026). The scoring scheme was deliberately designed to assign twice the value to specific markers of SA (sWBC count and synovial neutrophil percentage) compared to non-specific markers such as serum CRP and leukocyte count. These points are added to calculate the score (see Table 1).

**Table 1:** Criteria of the Septic Arthritis Evaluation Score (SAES).

SAES criterion	Threshold	Points
sWBC	>35650 cells/ µ L	2
Neutrophil percentage	>90.6 %	2
Serum CRP	>5 mg L^−1^	1
Leukocytes	<4 /nl or >12 /nl	1

### 2.4 Statistical analysis

Statistical analysis was performed with IBM SPSS version 28.0 (IBM SPSS Inc., Chicago, IL, USA). Descriptive statistics were generated for all variables. Normally distributed continuous variables are presented as the mean and standard deviation (SD), while non-normally distributed data are reported as the median and interquartile range (IQR). Categorical variables are summarized as absolute and relative frequencies. For group comparisons, Student's t test was used for normally distributed data, and the Mann–Whitney U test was used for non-normally distributed data. Statistical significance was defined as a two-sided p value < 0.05. We generated receiver-operating curves (ROCs) for sWBC, neutrophil percentage, and the Septic Arthritis Evaluation Score (SAES). Sensitivity and specificity were calculated for all points on the ROC curve. To determine the best cut-off, we applied the Youden index (sensitivity + specificity - 1) (Hajian-Tilaki, 2013; Gramlich et al., 2023).

Receiver-operating characteristic (ROC) analyses were used to assess the discriminatory ability of individual parameters and the composite score. The area under the curve (AUC) was interpreted descriptively as a measure of overall discrimination. No predefined threshold for clinical relevance was applied, and all results should be interpreted in the context of the exploratory nature of this retrospective analysis.

## 3 Results

### 3.1 Demographic data

During the study period, 133 cases of septic arthritis of native knees and shoulders were identified. A total of 88 patients were excluded due to incomplete diagnostic data, prior antibiotic therapy, or evidence of crystal-proven gout. Consequently, 45 patients were eligible for inclusion in the ROC analysis. The control group comprised 41 patients.

The knee was affected in 82.2 % of patients, and the shoulder was affected in 17.8 % of patients. The mean age of the patients was 62.5 years (SD ± 15.4), and most of them were male (68.9 %). Most patients were slightly overweight, with a median BMI of 27.8 (IQR 7.2); the highest BMI was 51. The majority of the patients had one or more medical co-morbidities (median CCI = 1.0, IQR = 4.0, range of 0–8), while 13 patients (28.9 %) had no previous illnesses. Overall, 66.7 % of the patients had an ASA score ≥ 3, indicating a cohort with substantial underlying health conditions.

Nine patients (20.0 %) had a documented history of previous surgery on the affected joint. However, due to the retrospective study design, detailed information on the exact surgical procedures performed and the presence of retained foreign material was not systematically available (Table 2).

**Table 2:** Demographic data of patients with SA of native knees and shoulders ( n=45 ).

Characteristics		Min.	Max.
Age (mean)	62.5 (SD ± 15.4)	22	88
Sex ( n , %)	m=31 (68.9 %)/ f=14 (31.1 %)		
BMI (median)	27.8 (IQR = 7.2)	17	51
CCI (median)	1.0 (IQR = 4.0)	0	8
Previous surgery	n=9 (20.0 %)		
ASA score ( n , %)			
I	5 (11.1 %)		
II	10 (22.2 %)		
III	23 (51.1 %)		
IV	7 (15.6 %)		

**Figure A1:**
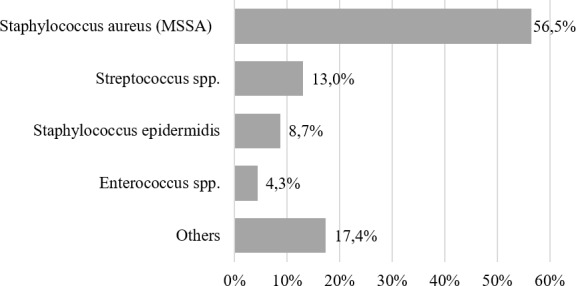
Causative pathogen in intraoperative biopsies. In 48.9 % no pathogen could be detected.

Microbiological and histopathological findings were not fully concordant in all cases. The microbiological analysis of preoperative joint fluid aspirates identified a causative pathogen in 16 cases (35.6 %). Intraoperative tissue cultures were positive in 23 patients (51.1 %). The most frequently detected organism was methicillin-sensitive *Staphylococcus aureus* (MSSA), accounting for 56.5 % of culture-positive cases (Fig. A1 in the Appendix). Histopathological examination revealed features consistent with infection in 37 patients (82.2 %). Notably, 13 patients (28.9 %) were culture-negative but showed histopathological findings typical of infection.

### 3.2 Laboratory markers and ROC analysis

In the laboratory analysis of patients with SA ( n=45 ), preoperative results showed a median serum CRP level of 158 mg L^−1^ and a serum leukocyte count of 11/nl. In comparison, the non-infected control group had a median serum CRP level of 72.1 mg L^−1^ and a leukocyte count of 9.1/nl.

In the joint fluid aspirates, patients with SA exhibited a median synovial WBC count of 42 910 cells/ µ L and a neutrophil percentage of 93.6 %. In contrast, the non-infected control group showed a median synovial WBC count of 9660 cells/ µ L and a neutrophil percentage of 86.8 % (Table 3).

**Table 3:** Laboratory markers of patients with SA compared to controls.

Laboratory markers	Infected ( n=45 )	Non-infected ( n=41)	P
			value
Serum CRP	158.0 mg L^−1^	72.1 mg L^−1^	<0.01
(median)	(IQR = 126.5, range 14.6–500.0)	(IQR = 117.8, range 2.2–268)	
Serum leukocyte	11.0/nl	9.1/nl	0.046
count (median)	(IQR = 5.5, range 3.3–38.7)	(IQR = 3.9, range 3.2–18.9)	
Synovial white blood	42 910/ µ L	9660/ µ L	<0.01
cell count (median)	(IQR = 48 895, range 1061–206 400)	(IQR = 29 944, range 44–43 430)	
Synovial neutrophil	93.6 %	86.8 %	<0.01
percentage (median)	(IQR = 3.9, range 63.1–99.3)	(IQR = 29.2, range 6.3–97.8)	

In the ROC analysis, synovial WBC count demonstrated moderate discriminatory ability for septic arthritis. A cut-off value of 35 650 cells/ µ L was associated with a sensitivity of 64.4 % and a specificity of 87.8 % (AUC 0.840, 95 % CI 0.759–0.920). Lower cut-off values were associated with higher sensitivity at the expense of specificity; for example, a threshold of 16 315 cells/ µ L yielded a sensitivity of 88.9 % and a specificity of 63.4 %. The previously reported cut-off by Massey et al. (2021) of 33 000 cells/ µ L showed comparable diagnostic characteristics within this cohort. For synovial neutrophil percentage, a cut-off of 90.6 % resulted in a sensitivity of 77.8 % and a specificity of 73.2 % (AUC 0.781, 95 % CI 0.683–0.879).

The composite Septic Arthritis Evaluation Score (SAES) demonstrated higher sensitivity than individual laboratory parameters in this dataset. A threshold of ≥3 points was associated with a sensitivity of 95.6 % and a specificity of 70.7 % (AUC 0.877, 95 % CI 0.801–0.953). All AUC values were statistically greater than 0.5 ( p<0.01 ), indicating discriminatory ability at the group level.

## 4 Discussion

This study was conducted to evaluate diagnostic laboratory parameters in native joint septic arthritis. Specifically, it aimed to assess synovial white blood cell count and neutrophil percentage cut-off values currently reported in the literature, which generally range between 20 000 and 50 000 cells/ µ L (Walinga et al., 2021) and 80 %–90 % neutrophils, respectively (Massey et al., 2021; Salazar et al., 2022). The results of the ROC analysis are shown in Fig. 2.

The findings of this study indicate that the previously published cut-off values of between 30 000 and 35 000 cells/ µ L serve as a useful reference range, while a widely cited threshold of 50 000 cells/ µ L applied to just 44 % of the patients in this cohort (Movassaghi et al., 2019; Salazar et al., 2022; McGillicuddy et al., 2007).

In the present analysis, two sWBC count cut-off values demonstrated comparable diagnostic performance, as reflected by identical Youden indices. While a lower threshold of 16 315 cells per/ µ L was associated with higher sensitivity, it also resulted in reduced specificity. Given that such low cut-off values are infrequently reported in the literature, this threshold was not incorporated into the SAES. A recent meta-analysis by Walinga et al. (2021) identified only a limited number of studies reporting comparably low cut-off values, with 50 000 cells/ µ L being the most commonly cited threshold (Walinga et al., 2021). Massey et al. reported a cut-off value of approximately 30 000 cells/ µ L in patients without prior antibiotic exposure (Massey et al., 2021).

For the purpose of score construction, the higher cut-off value of 35 650 cells/ µ L was therefore selected to reduce false-positive classifications and to maintain a balanced trade-off between sensitivity and specificity. This choice was pragmatic and data-driven and should be regarded to be exploratory.

The timing of diagnostic evaluation represents an important potential confounder in the interpretation of laboratory cut-off values as serum and synovial inflammatory parameters are known to increase with ongoing infection and treatment delay. In this retrospective cohort, the timing of joint aspiration and blood sampling relative to symptom onset was not standardized. Consequently, lower laboratory values may partly reflect early diagnostic assessment rather than reduced disease severity. This may have contributed to the comparatively low cut-off values observed in the present analysis and limits the generalizability of specific thresholds.

Previous studies have not consistently specified whether patients with gout or rheumatoid arthritis were considered to be differential diagnoses. To reduce bias and improve the validity of our results, such patients were excluded from the study in advance (Baillet et al., 2019; Luo et al., 2020). Crystal-induced arthritis represents a major differential diagnosis of septic arthritis as both conditions may present with similar clinical, biochemical, and synovial fluid findings. Although patients with crystal-proven arthritis were excluded, crystal analysis may yield false-negative results, and septic arthritis can coexist with gout or pseudogout. Consequently, misclassification cannot be fully excluded and reflects an inherent diagnostic challenge in the evaluation of suspected septic arthritis (Baillet et al., 2019; Papanicolas et al., 2012).

Septic arthritis caused by low-virulence organisms, such as coagulase-negative staphylococci, is uncommon in native joints without prior intervention but has been increasingly described following arthroscopic or intra-articular procedures. Comparative microbiological analyses have demonstrated distinct pathogen distributions between native and periprosthetic joint infections, with low-virulence organisms occurring more frequently in intervention-associated cases (Linke et al., 2009). Population-based data further indicate a rising incidence of postarthroscopic septic arthritis in native joints (Gunnlaugsdóttir et al., 2022). These infections may present with attenuated inflammatory responses and lower synovial cell counts, potentially contributing to diagnostic uncertainty and lower laboratory cut-off values.

To explore a composite scoring approach, the Septic Arthritis Evaluation Score (SAES) was constructed post hoc based on the results of the laboratory parameter evaluation in this cohort. The score incorporates four routinely assessed parameters: synovial white blood cell (sWBC) count, synovial neutrophil percentage, serum C-reactive protein (CRP), and serum leukocyte count. Parameter weighting was chosen to reflect the greater diagnostic relevance of joint-specific markers compared with non-specific systemic inflammatory markers. Accordingly, sWBC count and synovial neutrophil percentage were assigned higher weights than serum CRP and serum leukocyte count. A weighting of two points for synovial parameters and a maximum score of six points were selected as receiver-operating characteristic analyses did not indicate additional discriminatory benefit with higher weighting. The threshold of ≥3 points for the SAES was selected based on the ROC analysis as this cut-off yielded the highest Youden index within the present dataset. In this cohort, the SAES demonstrated higher sensitivity than individual laboratory parameters. The SAES should therefore be regarded as an exploratory tool, and its diagnostic performance requires validation in independent cohorts.

Since other diagnostic markers in synovial fluid, such as alpha-defensin and interleukin-6 (IL-6), have not demonstrated additional diagnostic value for periprosthetic joint infections or infections of native joints (Cooper et al., 2021), these markers were not included in this study or in the SAES calculation. The detection of calprotectin has demonstrated a strong ability to differentiate SA from pseudogout and rheumatoid arthritis in a recent study (Baillet et al., 2019). Nevertheless, it was not included in the calculation of the SAES as only routinely assessed parameters that are universally available in standard clinical laboratories were considered. Microbiological findings were also not incorporated into the SAES. Although microbiology remains essential for definitive diagnosis and targeted antimicrobial therapy, culture results are typically not available at the time of initial clinical decision-making and therefore lack value as a real-time diagnostic parameter. In addition, culture-negative septic arthritis is well described. Including microbiological results in the score would likely increase retrospective diagnostic accuracy but would reduce its applicability for early diagnostic evaluation, which was the primary intent of the score.

However, in this investigation, lower sensitivity and specificity values were observed compared to previous studies. Several factors may account for the observed discrepancies, especially compared to Massey et al. (2021). When analysing the cohort sizes, Massey et al. (2021) could include 44 patients with SA without previous antibiotic therapy for ROC analysis, and a control group of 257 patients without SA. Conversely, our cohort included 45 patients diagnosed with SA and a comparably sized control group of 41 patients without SA. Furthermore, our study population differed from other investigated study populations with higher ASA and CCI scores (Abram et al., 2020; Jung et al., 2018). Information on pre-existing conditions or ASA scores was not provided in the study by Massey et al. (2021).

### Limitations

This study has several important limitations that should be considered when interpreting the results. First, the retrospective design limits the control over data completeness, timing of diagnostic procedures, and standardization of the diagnostic work-up. As a result, a substantial proportion of 88 patients could not be included in the ROC analyses due to missing laboratory data, which may have introduced selection bias.

Second, the sample size of the ROC analysis cohort was limited. Although comparable to previous studies evaluating synovial WBC count cut-off values, the relatively small number of cases restricts the precision and stability of estimated cut-off values and AUCs. Consequently, small changes in the dataset may have resulted in relevant shifts in diagnostic thresholds.

Third, the diagnostic definition of septic arthritis relied on microbiological and/or histopathological findings. Histopathological assessment was qualitative and not based on a validated or standardized scoring system, which may have introduced diagnostic uncertainty. Furthermore, microbiological and histopathological findings were not fully concordant in all cases, underscoring the absence of a definitive reference standard.

Fourth, clinical signs and symptoms were deliberately not incorporated into the diagnostic definition to ensure objectivity and reproducibility. While this approach is consistent with established definitions for periprosthetic joint infection, it may limit the applicability of the findings to real-world clinical decision-making, where clinical presentation remains essential.

Fifth, the SAES was constructed post hoc and evaluated within the same dataset from which its parameters and weighting were derived. The score should therefore be regarded to be exploratory and hypothesis-generating. Its diagnostic performance may be overestimated, and external validation in independent and prospectively collected cohorts is required before any clinical application can be considered. In addition, relevant false-negative and false-positive classifications were observed when applying individual laboratory cut-off values. A total of 13 of the 45 patients in the ROC analysis fulfilled the diagnostic definition of septic arthritis despite having an sWBC count below the selected cut-off value. Conversely, five patients were classified as non-infected despite sWBC counts above the cut-off. Notably, four of these patients also showed synovial neutrophil percentages above the corresponding threshold and high SAES values. These findings highlight the substantial overlap of laboratory parameters between infected and non-infected patients and underscore the limitations of relying on isolated cut-off values for individual patient classification.

Sixth, the analysis was restricted to septic arthritis of native knee and shoulder joints. The diagnostic performance of the evaluated laboratory parameters and the SAES may differ in other native joints, and the results should not be directly extrapolated to infections of other joint locations.

Finally, the study was conducted at a single tertiary referral centre, which may limit the generalizability of the findings to other clinical settings, particularly smaller hospitals with different patient populations and diagnostic workflows.

## 5 Conclusion

This study evaluated commonly used laboratory parameters for the diagnosis of septic arthritis of native knee and shoulder joints and explored a composite laboratory-based scoring approach. Individual parameters showed limited discriminatory ability when applied in isolation, with substantial overlap between infected and non-infected patients. The Septic Arthritis Evaluation Score (SAES), which combines synovial and serum markers, demonstrated higher sensitivity within this retrospective cohort. However, the score was constructed post hoc, and its diagnostic performance is exploratory. Prospective validation in larger and independent cohorts is required before any clinical application can be considered.

## Data Availability

The software code and data supporting the conclusions of this article can be made available by the authors upon reasonable request.
